# Protective Effect of Degraded *Porphyra yezoensis* Polysaccharides on the Oxidative Damage of Renal Epithelial Cells and on the Adhesion and Endocytosis of Nanocalcium Oxalate Crystals

**DOI:** 10.1155/2021/6463281

**Published:** 2021-03-03

**Authors:** Qian-Long Peng, Chuang-Ye Li, Yao-Wang Zhao, Xin-Yuan Sun, Hong Liu, Jian-Ming Ouyang

**Affiliations:** ^1^Department of Urology, Hunan Children's Hospital, Changsha 410007, China; ^2^Institute of Biomineralization and Lithiasis Research, Jinan University, Guangzhou 510632, China

## Abstract

The protective effects of *Porphyra yezoensis* polysaccharides (PYPs) with molecular weights of 576.2 (PYP1), 105.4 (PYP2), 22.47 (PYP3), and 3.89 kDa (PYP4) on the oxidative damage of human kidney proximal tubular epithelial (HK-2) cells and the differences in adherence and endocytosis of HK-2 cells to calcium oxalate monohydrate crystals before and after protection were investigated. Results showed that PYPs can effectively reduce the oxidative damage of oxalic acid to HK-2 cells. Under the preprotection of PYPs, cell viability increased, cell morphology improved, reactive oxygen species levels decreased, mitochondrial membrane potential increased, S phase cell arrest was inhibited, the cell apoptosis rate decreased, phosphatidylserine exposure reduced, the number of crystals adhered to the cell surface reduced, but the ability of cells to endocytose crystals enhanced. The lower the molecular weight, the better the protective effect of PYP. The results in this article indicated that PYPs can reduce the risk of kidney stone formation by protecting renal epithelial cells from oxidative damage and reducing calcium oxalate crystal adhesion, and PYP4 with the lowest molecular weight may be a potential drug for preventing kidney stone formation.

## 1. Introduction

A kidney stone is a complex multifactorial disease and one of the common causes of renal damage. Exposure of renal epithelial cells to high oxalic acid can induce oxidative stress of cells and generate reactive oxygen species (ROS), thereby causing oxidative damage to renal epithelial cells and inducing the formation of kidney stones [1, 2]. Therefore, finding low-cost and effective drugs to reduce the damage of renal epithelial cells caused by oxalic acid is important to prevent kidney stones.

Studies over the past decades have shown that antioxidants in diet may help prevent or delay oxidative damage, thereby reducing the risk of various diseases caused by oxidative damage [3]. Seaweed polysaccharides are natural biological compounds that are beneficial to human health due to their effective antioxidant properties and free radical scavenging properties [4–6]. Presa et al. [7] obtained six kinds of sulfated polysaccharides from green seaweed *Udotea flabellum*, which can protect cells from oxidative damage caused by FeSO_4_, CuSO_4_, and ascorbate.

The factors that affect the activity of polysaccharides include the acid group content, polysaccharide structure, polysaccharide conformation, and molecular weight (Mw) of polysaccharides [8]. Di Lorenzo et al. [9] showed that polysaccharides from *Opuntia ficus* can repair wounds and accelerate skin regeneration, and polysaccharides with low Mw have strong repair activity. Sun et al. [10] proved that low-Mw polysaccharides from *Porphyridium cruentum* (6.53 kDa) have better antitumor and immunoregulatory activities than high-Mw polysaccharides (903.3 kDa). Zhao et al. [11] showed that tea polysaccharides (TPS0, TPS1, TPS2, and TPS3) with Mws of 10.88, 8.16, 4.82, and 2.3 kDa, respectively, could inhibit the adhesion of calcium oxalate monohydrate (COM) to human renal proximal tubular (HK-2) cells, and TPS2 with moderate Mw had the best protective effect. In addition, TPS with lower molecular weight have a better ability to increase the percentage of the dihydrate crystalline phase in CaOx crystals and reduce the size of CaOx monohydrate crystals [12].

The *Porphyra yezoensis* polysaccharide (PYP) is a low-cost, rich-source polysaccharide with high sulfuric acid groups and has good antioxidant capacity. PYP has a typical *Porphyran* structure and a backbone of alternating (1 → 3)-linked *β*-D-galactose units and (1 → 4)-linked 3,6-anhydro-*α*-L-galactose or (1 → 4)-linked *α*-L-galactose 6-sulfate units [13]. Qian et al. [14] showed that plasma triglycerides, total cholesterol, and plasma low-density lipoprotein cholesterol in rats after oral administration of PYP were significantly reduced, indicating that PYP had a hypolipidemic activity. Fu et al. [15] proved that PYP has an immunoregulatory activity. PYP regulates the immune system by activating the NF-*κ*B signaling pathway, promotes differentiation and activation of regulatory T cells (Tregs), and regulates the balance of T helper (Th)1/Th2 cells to maintain immune homeostasis. Chen and Xue [16] demonstrated that PYPs can inhibit the proliferation of SGC-7901 tumor cells, induce tumor cell apoptosis, and have antitumor effects on SGC-7901 mice, which can be used for cancer prevention and treatment.

In previous studies [13], we degraded the original PYP with Mw = 4669 kDa, obtained a series of degraded polysaccharides with low Mw, and characterized their chemical structures. This PYP has a repair effect on oxidatively damaged HK-2 cells, and the polysaccharide with the lowest Mw (4.02 kDa) has the best repair effect. In addition, we also studied the differences in toxicity and calcification of hydroxyapatite (HAP) on A7R5 cells before and after PYP protection [17]. PYPs could effectively reduce the cytotoxicity of HAP and inhibit the osteogenic transformation of the A7R5 cells. PYP protection inhibited cell necrosis and decreased alkaline phosphatase activity and expressions of bone/chondrocyte phenotype genes.

The repair of damaged cells by polysaccharides is equivalent to the treatment of diseases, whereas the preprotection of cells from oxidative damage is equivalent to the active prevention of diseases. Therefore, the preprotection of cells with exogenous antioxidants is more important to human health than repairing damaged cells. Kidney stones not only cause great harm to human beings but also lead to serious economic losses. Using scientific methods to prevent the occurrence of kidney stones or reduce their recurrence rate will be less than the cost of treatment and will reduce the pain of patients. Therefore, clinically, the scientific significance of preventing kidney stones is greater than that of treatment. PYPs also have a protective effect from calcium oxalate crystals [18]. PYP protection reduced the crystal toxicity, prevented the destruction of the cytoskeleton, reduced the expression of osteopontin and transmembrane protein (CD44), and finally inhibited the adhesion and endocytosis of HK-2 cells to nano-COM.

Besides calcium oxalate crystals, oxalic acid is also one of the most common foreign toxic substances that lead to stone formation. But the protective effect of degraded PYPs on HK-2 cells from cytotoxicity of oxalic acid has not been studied. In this study, normal HK-2 cells were preprotected by PYPs with different Mws, and then we studied the resistance of the protected cells to oxidative damage of oxalic acid and the ability to inhibit crystal adhesion and promote crystal endocytosis. We aimed to provide insight into preventing kidney stone formation and delaying its recurrence.

## 2. Materials and Methods

### 2.1. Materials and Apparatus

#### 2.1.1. Materials

Natural *P*. *yezoensis* polysaccharide (PYP0) was provided by Shaanxi Ciyuan Biotechnology Co., Ltd. The polysaccharide content was 95%. Four different molecular weights of degraded polysaccharides PYP1 (576.2 kDa), PYP2 (105.4 kDa), PYP3 (22.47 kDa), and PYP4 (3.89 kDa) were obtained by the H_2_O_2_ oxidative degradation method [13]. The polysaccharide structure was characterized by ^1^H NMR, ^13^C NMR, FT-IR, and GC-MS spectral analysis. PYP has a typical *Porphyran* structure and has a backbone of alternating (1 → 3)-linked *β*-D-galactose units and (1 → 4)-linked 3,6-anhydro-*α*-L-galactose or (1 → 4)-linked *α*-L-galactose 6-sulfate units. The contents of the sulfate group (–OSO_3_H) of PYPs are 17.51% to 17.86%, and the contents of –COOH are 1.42% to 1.70% [13].

Calcium oxalate monohydrate (COM) crystals have a size of about 100 nm. Human proximal tubular epithelial (HK-2) cells were purchased from the Shanghai Cell Bank of the Chinese Academy of Sciences (Shanghai, China). The following materials were also purchased for the study: fetal bovine serum and cell culture medium (DMEM) (Gibco, USA); penicillin and streptomycin (Beijing Pubo Biotechnology Co., Ltd., Beijing, China); cell proliferation assay kit (CCK-8), JC-1 (5,5,6,6-tetrachloro-1,1′,3,3′-tetraethyl-imidacarbocyanine iodide) dye, Annexin V-FITC/PI cell apoptosis and necrosis double dye kits, 4′,6-diamidino-2-phenylindole (DAPI), fluorescein isothiocyanate (FITC), LysoTracker Red, and reactive oxygen species (ROS) kit (Shanghai Beyotime Bio-Tech Co., Ltd., Shanghai, China); and cell culture plates of 6, 12, and 96 wells (NEST, China). Conventional reagents such as oxalic acid and anhydrous ethanol are analytically pure (Guangzhou Chemical Reagent Factory, China).

#### 2.1.2. Apparatus

The apparatus used in the study include the following: microplate reader (Safire2, Tecan, Männedorf, Switzerland); X-L type environmental scanning electron microscope (SEM, Philips, Eindhoven, Netherlands); X-ray powder diffractometer (D/MAX2400, Japan); inverted fluorescence microscope (Leica DMRA2, Germany); optical microscope (Olympus, CKX41, Japan); flow cytometer (FACSAria, BD Corporation, CA, USA); and confocal laser scanning microscope (LSM510 Meta Duo Scan, Zeiss, Germany).

### 2.2. Experimental Methods

#### 2.2.1. Cell Culture and Grouping

HK-2 cells were cultured in DMEM containing 10% fetal bovine serum in a 5% CO_2_ humidified environment at 37°C. Upon reaching a monolayer of 80%–90% confluence, cells were gently blown after trypsinization to form a cell suspension for subsequent cell experiments. The cell suspension (1 × 10^5^ cells/mL) was inoculated per well in 96-well plates and incubated for 24 h. The cells were divided into three groups: (1) control group, where only the serum-free DMEM culture medium was added; (2) protection group, where the serum-free medium containing PYPs with concentrations of 20, 40, 60, 80, and 100 *μ*g/mL was added, and the culture medium was aspirated after 12 h. The cells were then treated with 2.8 mmol/L of oxalate dissolved in PBS and incubated for 3.5 h; and (3) injured group, in which 2.8 mmol/L of oxalate dissolved in PBS was added and incubated for 3.5 h.

#### 2.2.2. Cell Viability Detection

The density of seeded cells and experimental grouping were the same as those in Section 2.2.1. After the treatment time was reached, 10 *μ*L of CCK-8 reagent was added to each well and incubated for 1.5 h in the dark. Absorbance (*A*) was measured at 450 nm according to the CCK-8 kit instruction. Cell viability was determined using the following equation:

(1) $$\begin{array}{c} \text{Cell viability }\left(\%\right)=\frac{A\left(\text{treatment group}\right)}{A\left(\text{control group}\right)}\times 100. \end{array}$$

#### 2.2.3. Cell Morphology Observation by Hematoxylin-Eosin (HE) Staining

The cell suspension (1 × 10^5^ cells/mL, 2 mL) was inoculated per well in 6-well plates and incubated for 24 h. The cells were divided into three groups: (1) control group, where only the serum-free DMEM culture medium was added; (2) protection group, where the serum-free medium containing PYPs with concentrations of 100 *μ*g/mL was added, and the culture medium was aspirated after 12 h. The cells were then treated with 2.8 mmol/L of oxalate dissolved in PBS and incubated for 3.5 h; and (3) injured group, in which 2.8 mmol/L of oxalate dissolved in PBS was added and incubated for 3.5 h. After the treatment time was reached, cells were fixed with 4% paraformaldehyde for 15 min and stained with hematoxylin and eosin according to the manufacturer's instructions. Morphological changes of the cells were observed under a microscope.

#### 2.2.4. Changes in Intracellular Reactive Oxygen Species (ROS) Levels

We followed the methods of Huang et al. [17]. The density of seeded cells and experimental grouping were the same as those in Section 2.2.3. After the treatment time was reached, 500 *μ*L of DCFH-DA diluted with the serum-free medium was added. The samples were stained with DCFH-DA for 30 min, the cells were qualitatively observed under a fluorescence microscope, and the fluorescence intensity was quantitatively detected by a microplate reader.

#### 2.2.5. Measurement of Mitochondrial Membrane Potential (ΔΨm)

We followed the methods of Huang et al. [17]. The density of seeded cells and experimental grouping were the same as those in Section 2.2.3. After the treatment time was reached, the cells were stained with JC-1 for 1 h in the dark, and the cells were qualitatively observed under a fluorescence microscope. In the same operation as above, the cells were digested with 0.25% trypsin (moderate digestion), and the cell deposits were centrifuged at 1000 rpm for 5 min. The samples were detected by flow cytometry according to the requirements of the JC-1 kit.

#### 2.2.6. Cell Cycle Detection

The density of seeded cells and experimental grouping were the same as those in Section 2.2.3. After the treatment time was reached, the cells were washed twice with PBS and digested with 0.25% trypsin; then, 10% fetal bovine serum DMEM was used to terminate digestion, and the cells were centrifuged at 1000 rpm for 5 min. The collected cells were washed twice with PBS, then fixed using 70% ethanol for 24 h at 4°C. Ethanol was removed by centrifugation, and the cells were washed twice with PBS. Cells were then resuspended in 200 *μ*L propidium iodide (PI) and kept at 37°C for 15 min. The cell cycle was analyzed by the flow cytometer.

#### 2.2.7. Apoptosis and Necrosis Detection

We followed the methods of Huang et al. [17]. The density of seeded cells and experimental grouping were the same as those in Section 2.2.3. After the treatment time was reached, the cells were washed twice with PBS and digested with 0.25% trypsin; then, 10% fetal bovine serum DMEM was used to terminate digestion, and the cells were centrifuged at 1000 rpm for 5 min. Then, 200 *μ*L of the binding buffer was added and mixed thoroughly into the cells. The cells were stained with 5 *μ*L of Annexin V-FITC, incubated in the dark at room temperature for 10 min, and then centrifuged. The supernatant was removed. Afterward, 200 *μ*L of the binding buffer was added and mixed thoroughly into the cells. Then, 5 *μ*L of PI was added to stain the cells. After the treatment was administered, the cells were detected through flow cytometry.

#### 2.2.8. Phosphatidylserine (PS) Exposure Assay

The density of seeded cells and experimental grouping were the same as those in Section 2.2.3. After the treatment time was reached, the cells were washed twice with PBS and digested with 0.25% trypsin; then, 10% fetal bovine serum DMEM was used to terminate digestion, and the cells were centrifuged at 1000 rpm for 5 min. 200 *μ*L of the binding buffer was added and mixed thoroughly, stained with 5 *μ*L of Annexin V-FITC for 30 min at 4°C in the dark, and then detected through flow cytometry.

#### 2.2.9. Preparation of Fluorescence-Labeled COM

A mixture of 0.05 g of COM and 5 mL of APTES in 50 mL of anhydrous ethanol was refluxed with continuous stirring under nitrogen for 3 h. Next, 0.025 g of FITC was added to the mixture for a reaction time of 6 h at 74°C. The FITC-tagged COM was then collected through centrifugation, washed several times with anhydrous ethanol and distilled water to ensure that no free FITC remained, and dried.

#### 2.2.10. Quantitative Analysis of Internalized COM Crystals

We followed the methods of Huang et al. [17]. The density of seeded cells and experimental grouping were the same as those in Section 2.2.3. Cells in groups were exposed to a serum-free medium containing 200 *μ*g/mL FITC-labeled COM crystals for 24 h. After reaching the incubation times, the cells were treated with ethylenediaminetetraacetic acid (EDTA) (5 mM) for 5 min to remove the adherent COM, and the percentages of cells with internalized crystals and the fluorescence intensity were measured by flow cytometry.

#### 2.2.11. Observation of COM Localization in Lysosomes

We followed the methods of Huang et al. [17]. The density of seeded cells and experimental grouping were the same as those in Section 2.2.3. Cells in groups were exposed to a serum-free medium containing 200 *μ*g/mL FITC-labeled COM crystals for 24 h. After reaching the incubation times, the cells were stained with 70 nM LysoTracker Red to label lysosomes for 2 h and then fixed with paraformaldehyde for 30 min, and the cell nucleus was stained with DAPI. The crystal distribution was observed by a confocal laser scanning microscope.

#### 2.2.12. Quantitative Analysis of Adherent COM Crystals

We followed the methods of Huang et al. [17]. The density of seeded cells and experimental grouping were the same as those in Section 2.2.3. Cells in groups were exposed to a serum-free medium containing 200 *μ*g/mL FITC-labeled COM crystals for 1 h in 4°C that inhibited endocytosis of cells to COM [19]. The cells were washed twice with cold PBS to eliminate unbound crystals, followed by trypsinization, and the percentage of cells with adherent crystals was measured by a flow cytometer.

#### 2.2.13. SEM Observation of Adhered Crystals on the Cell Surface

The density of seeded cells and experimental grouping were the same as those in Section 2.2.3. Cells in groups were exposed to a serum-free medium containing 200 *μ*g/mL COM crystals for 1 h. After reaching the adhesion time, the cells were washed with PBS and fixed in 2.5% glutaraldehyde at 4°C for 24 h, dehydrated in gradient ethanol (30%, 50%, 70%, 90%, and 100%, respectively), dried under the critical point of CO_2_, and treated with gold sputtering. The crystal adhesion was observed by SEM.

#### 2.2.14. Statistical Analysis

Experimental data were expressed as the mean ± standard deviation ($\bar{x}\pm \text{SD}$). The experimental results were analyzed statistically using SPSS 13.0 software (SPSS Inc., Chicago, IL, USA). The differences in the means between the experimental groups and the control group were analyzed using one-way ANOVA, followed by the Tukey post hoc test. If *p* < 0.05, there was significant difference; if *p* < 0.01, the difference was extremely significant; if *p* > 0.05, there was no significant difference.

## 3. Results

### 3.1. PYP Preprotection Reduces Oxalic Acid Damage to HK-2 Cells

**Figure 1:**
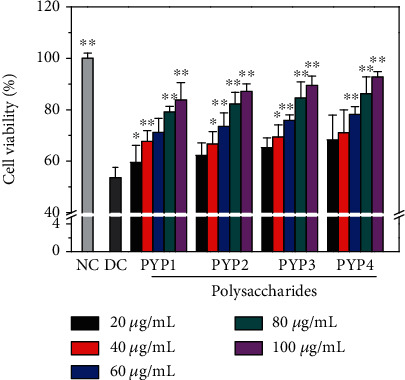
Cell viability change of HK-2 cells before and after PYP protection. NC: normal control; DC: damaged control. Oxalate damage concentration: 2.8 mmol/L; damage time: 3.5 h; protective time: 12 h. Compared with the DC group, ^∗^*p* < 0.05, ^∗∗^*p* < 0.01.

Four kinds of PYPs were used to preprotect HK-2 cells. The oxidative damage of oxalic acid to HK-2 cells before and after PYPs preprotected cells was detected by the CCK-8 method (Figure 1). The cell viability of the injured group (53.68%) was significantly lower than that of the control group (100%), whereas the cell viability of the protection group was higher than that of the injured group, indicating that the preprotection of the four PYPs could improve the ability of HK-2 cells to resist oxidative damage caused by oxalic acid.

As the Mw of PYP decreased or the concentration of polysaccharides increased, the viability of cells increased; that is, the low-Mw PYP4 had a good preprotective effect. At a concentration of 100 *μ*g/mL, the cell viability after 12 h preprotection by PYP1, PYP2, PYP3, and PYP4 was 83.88%, 87.21%, 89.43%, and 92.84%, respectively.

### 3.2. PYP Preprotection Improves Cell Morphology

**Figure 2:**
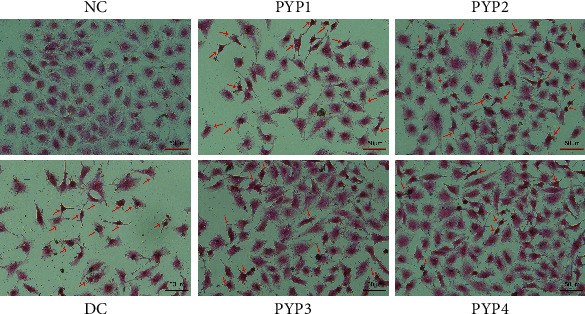
Morphological changes of HK-2 cells before and after PYP protection. *c*(PYP) = 100 *μ*g/mL; oxalate damage concentration: 2.8 mmol/L; damage time: 3.5 h; protective time: 12 h. The red arrow indicates apoptotic cells.

Hematoxylin-eosin staining was used to observe the morphological changes of damaged HK-2 cells before and after PYP protection (Figure 2). Normal HK-2 cells were tightly connected, large, and plump. The morphology of HK-2 cells in the injured group was irregular and disordered, the cell volume was reduced, the cell density was reduced, and the cells had the tendency to apoptosis. HK-2 cells preprotected by PYPs were less damaged by oxalic acid, resulting in the increase in the number of normal cells and decrease in the number of apoptotic cells (indicated by arrows in Figure 2), of which PYP4 had the best protective effect, indicating that PYP4 with low Mw had the best protective ability for HK-2 cells.

### 3.3. PYP Preprotection Reduces Intracellular ROS

**Figure 3:**
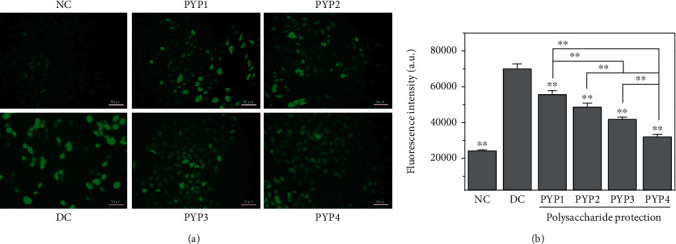
ROS changes in HK-2 cells before and after PYP protection. (a) Fluorescence microscopy images. (b) Quantitative fluorescence intensity of ROS. NC: normal control. DC: damaged control. Oxalate damage concentration: 2.8 mmol/L; damage time: 3.5 h; *c*(PYP) = 100 *μ*g/mL; protective time: 12 h. Compared with the DC group, ^∗^*p* < 0.05, ^∗∗^*p* < 0.01.

Figure 3 shows the changes in the ROS of cells after protecting PYPs. Compared with the normal group, HK-2 cells in the injured group have the strongest fluorescence intensity, indicating that the ROS level was the highest. However, the fluorescence intensity of ROS in the cells preprotected by PYPs decreased to different degrees, indicating that PYPs can reduce the generation of ROS in the cells, thereby reducing the oxidative damage of the cells, and PYP4 with the lowest Mw had the most significant degree of reduction.

### 3.4. PYP Preprotection Inhibits Decline in Mitochondrial Membrane Potential (ΔΨm)

**Figure 4:**
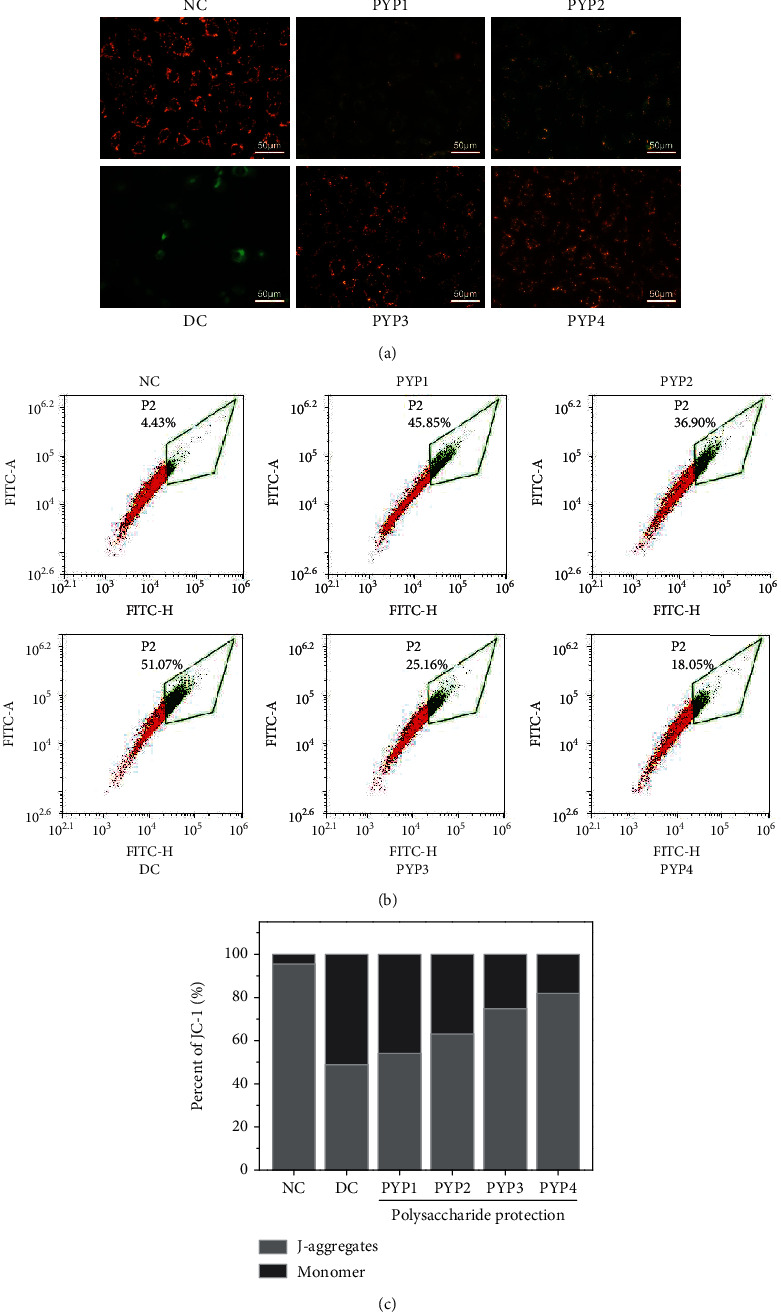
ΔΨm changes in HK-2 cells before and after PYP protection. (a) Fluorescence microscopy images. (b) Flow cytometric data of ΔΨm. (c) Quantitative results. Experimental conditions and statistical significance are the same as in Figure 3.

The fluorescent probe JC-1 is a cationic lipophilic dye that can freely pass through the cell membrane. At high ΔΨm (normal cells), the fluorescent probe JC-1 molecules accumulated in the mitochondrial matrix to form polymers (J-aggregates), which produced red fluorescence (Figure 4(a)). When cells are damaged, ΔΨm decreased, and JC-1 was primarily in a monomer form (JC-1-monomer), producing green fluorescence.

Figure 4(a) is the change of ΔΨm quantitatively detected by flow cytometry. Compared with the normal group of cells (4.43%), the red fluorescence of the injured cells was weaker, and the green fluorescence was stronger, indicating that ΔΨm decreased remarkably (51.07%). After the protection of PYPs, the red fluorescence of the cells increased, the green fluorescence decreased, ΔΨm decreased in different degrees, and the decreased value of ΔΨm decreased (18.05%–45.85%) (Figure 4(c)), indicating that the degree of cell damage was reduced.

### 3.5. S Phase Cells Decreased and G1 Phase Cells Increased after PYP Preprotection

**Figure 5:**
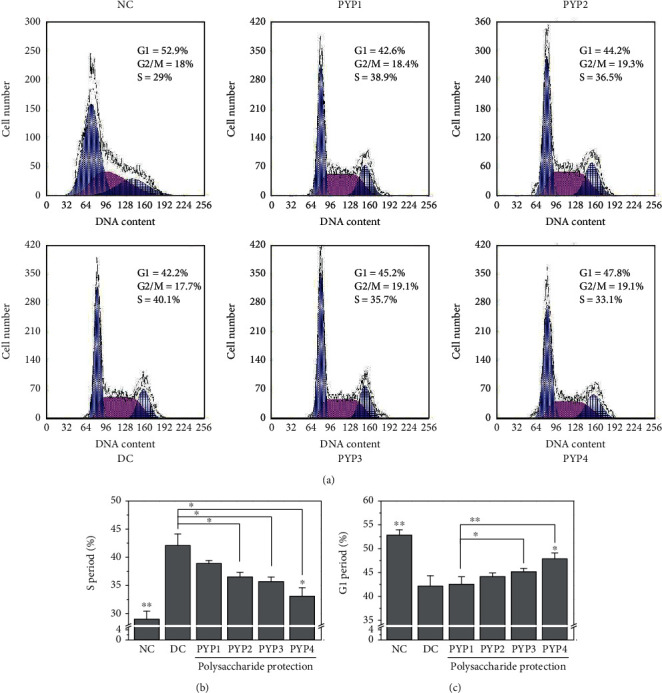
Cell cycle progressions of HK-2 cells before and after PYP protection. (a) Flow cytometric data of the cell cycle. (b) Quantitative result of the S phase. (c) Quantitative result of the G1 phase. Experimental conditions and statistical significance are the same as in Figure 3.

The stagnation of the cell cycle reflects the degree of DNA damage. The greater the degree of DNA damage, the greater the cell damage. After the normal HK-2 cells were damaged by oxalic acid, the number of cells in the S phase increased (Figure 5(b)), and the number of cells in the G1 phase decreased (Figure 5(c)), indicating that oxalic acid-damaged HK-2 cells were arrested in the S phase. After PYP preprotection, S phase cells decreased and G1 phase cells increased, among which the number of G1 phase cells preprotected by PYP4 increased more, indicating that PYP4 had a better protective effect.

### 3.6. PYP Preprotection Inhibits Cell Apoptosis

**Figure 6:**
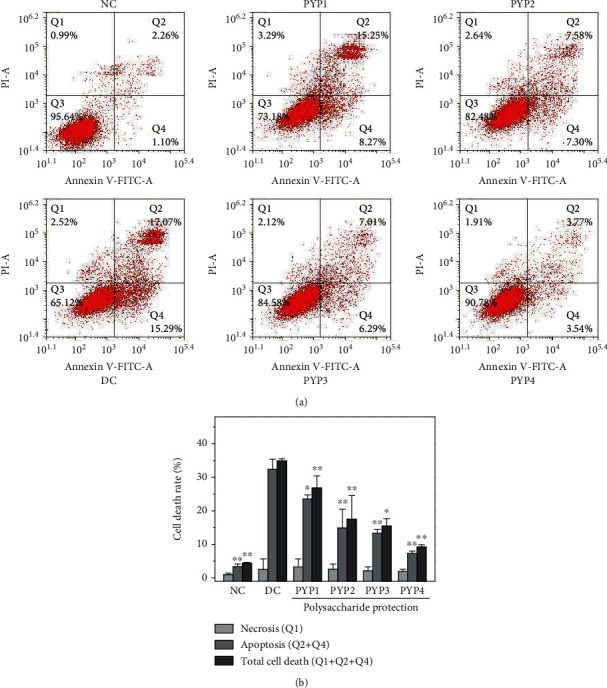
Cell apoptosis and necrosis of HK-2 cells before and after PYP protection. (a) Flow cytometric data of cell apoptosis and necrosis. (b) Quantitative results. Experimental conditions and statistical significance are the same as in Figure 3.

Figure 6 shows apoptosis and necrosis of cells before and after PYP preprotection. The result was quantitatively detected by the Annexin V/PI double staining method. Compared with the control group (apoptotic cells (3.36%)), the number of apoptotic cells (32.36%) in the oxalic acid-damaged group was significantly increased. After PYP protection, PYPs can effectively reduce the number of apoptotic cells (7.31%–23.52%), of which PYP4 had a better inhibitory effect (7.31%).

### 3.7. PYP Preprotection Inhibits Phosphatidylserine (PS) Exposure

**Figure 7:**
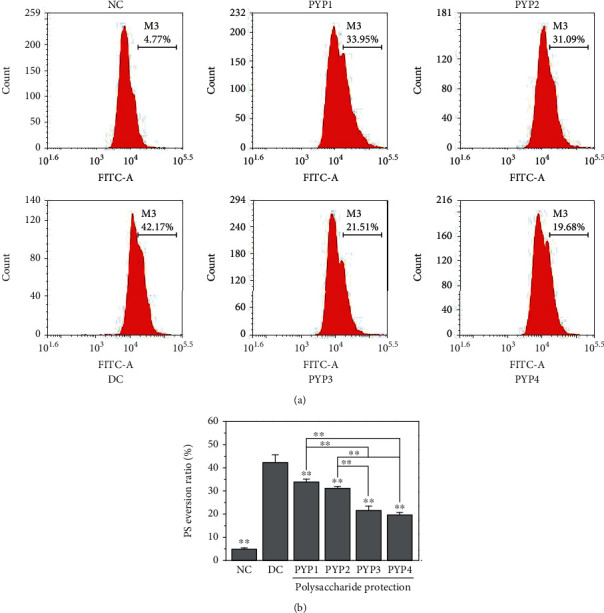
PS exposure of HK-2 cells before and after PYP protection. (a) Flow cytometric data of PS exposure. (b) Quantitative results. Experimental conditions and statistical significance are the same as in Figure 3.

Under normal circumstances, PS is located inside the renal tubular epithelial cell membrane, but after the cell membrane is damaged, part of the PS will migrate to the outside of the cell membrane. As shown in Figure 7, the amount of PS exposure on the surface of HK-2 cells in the normal group was lower (4.77%), whereas the amount of PS exposure (42.17%) in the cells damaged by oxalic acid was significantly increased. After preprotection by PYPs, the amount of PS exposure decreased (19.68%–3.95%), and the protective effect of PYP4 was more significant.

### 3.8. PYP Preprotection Promotes Endocytosis of COM Crystals by Cells

**Figure 8:**
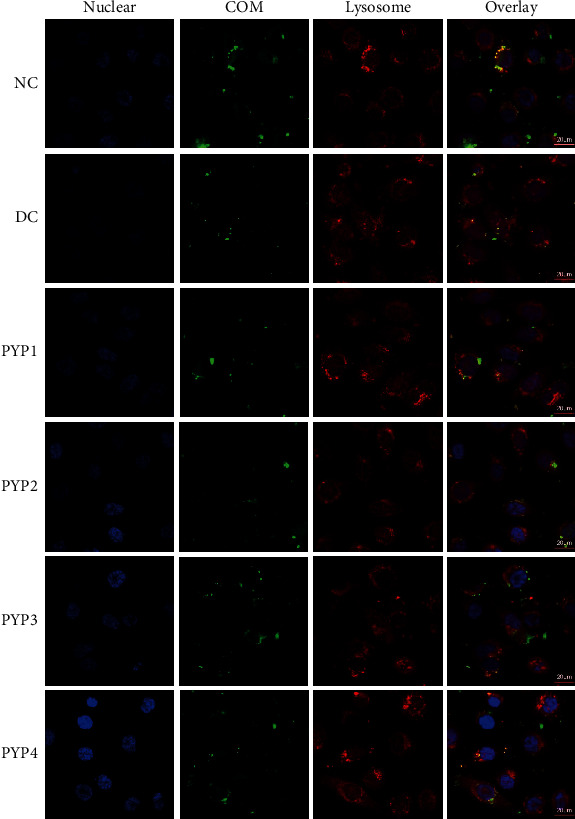
Observation of the accumulation of nano-COM in HK-2 cell lysosomes before and after PYP protection by confocal microscopy. COM concentration: 200 *μ*g/mL; endocytosis time: 24 h. Use DAPI and LysoTracker Red to label the nucleus (blue) and lysosome (red), respectively. Other experimental conditions are the same as in Figure 3.

After using DAPI and LysoTracker Red to label the nucleus and lysosome, respectively, the nucleus and lysosome showed blue fluorescence and red fluorescence, respectively. The distribution of green fluorescence-labeled COM crystals in cell lysosomes can be observed by a confocal microscope. As shown in Figure 8, the COM crystal with green fluorescence overlaps with the lysosome with red fluorescence, which indicated that the nano-COM crystal has entered the lysosome. A large number of COM crystals overlapped in normal cells indicating that more crystals were endocytosed by normal cells. However, the number of overlapping COM crystals in the injured group decreased, indicating that the damaged cell lysozyme has a reduced ability to internalize COM crystals. After the cells were preprotected by PYPs, the number of crystals entering the lysosome increased compared with the injured group, and as the Mw of PYP decreased, the number of crystals entering the lysosome gradually increased.

**Figure 9:**
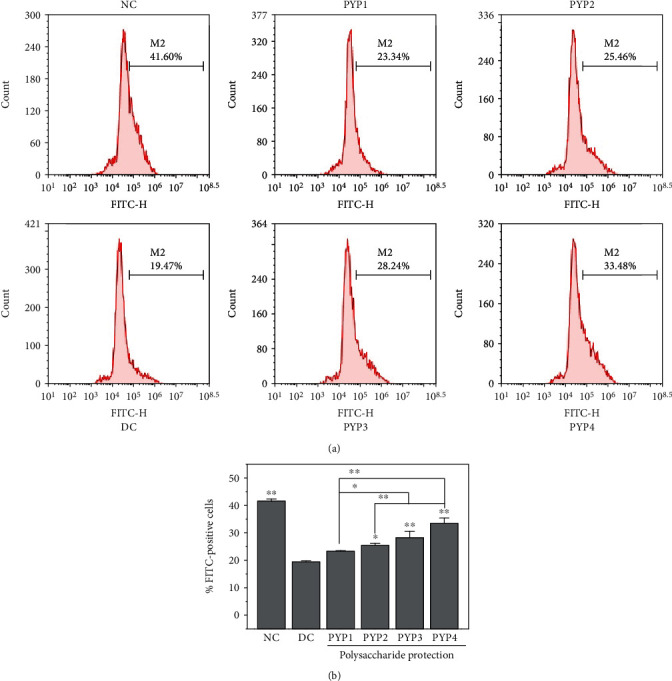
Flow cytometry analysis of internalized COM crystals by HK-2 cells before and after PYP protection. (a) Flow cytometric data. (b) Quantitative results. COM concentration: 200 *μ*g/mL; endocytosis time: 24 h. Use DAPI and LysoTracker Red to label the nucleus (blue) and lysosome (red), respectively. Compared with the DC group, ^∗^*p* < 0.05, ^∗∗^*p* < 0.01.

Flow cytometry was used to quantitatively detect the difference in endocytosis of nano-COM crystals by cells before and after PYP preprotection (Figure 9(a)). After incubating FITC-labeled COM crystals with a size of approximately 100 nm with HK-2 cells at 37°C for 24 h, EDTA was used to remove the crystals adhered to the cell surface. The cells with FITC signals detected were the cells that endocytosed crystals [20, 21]. The proportion of cells endocytosing crystals in the oxalic acid-damaged group (19.47%) was significantly lower than that in the normal control group (41.06%). The cell proportion of endocytic crystals after PYP preprotection was between the control and injured groups (23.34%–33.48%) (Figure 9(b)), and the lower the Mw, the larger the cell proportion of endocytic crystals.

### 3.9. PYP Preprotection Inhibits COM Adhesion on the Cell Surface

**Figure 10:**
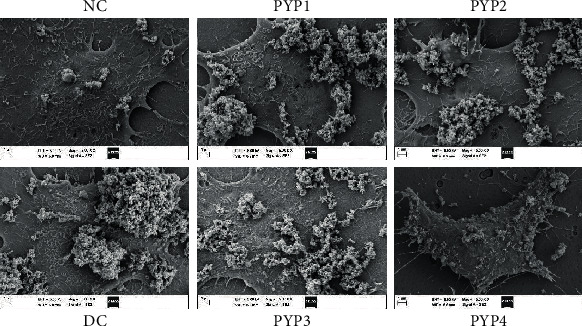
SEM observation of the nano-COM crystals adhered to the surface of HK-2 cells before and after PYP protection. COM concentration: 200 *μ*g/mL; adhesion time: 1 h.

A scanning electron microscope was used to observe the adhesion of HK-2 cells to COM crystals before and after PYP protection (Figure 10). The normal control group had plump cell morphology, smooth cell surface, and fewer amounts of adhered crystals. However, in the injured group, the cell morphology changed evidently, the cell surface became rough, and the amount of adherent crystals on the cell surface increased remarkably. The cells preprotected by PYPs were less damaged; the amount of crystals adhering to the cell surface was between the cells of the control and damaged groups, and as the Mw of PYP decreased, the amount of crystals gradually decreased; i.e., PYP4 with the lowest Mw had the best protective effect on the cells.

**Figure 11:**
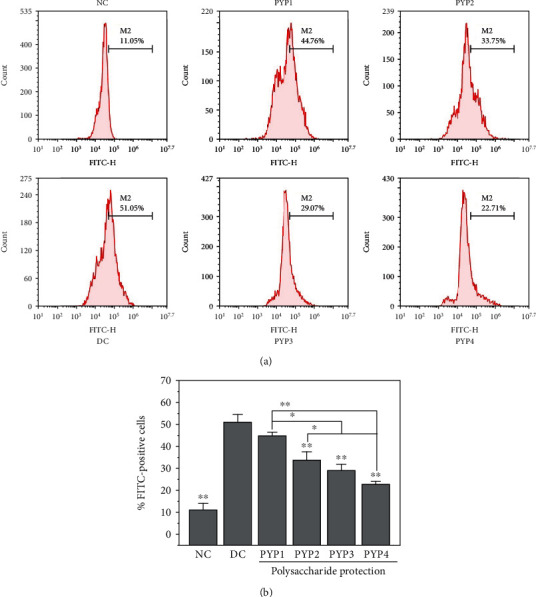
Flow cytometry quantitative analysis of adherent COM crystals on HK-2 cells before and after PYP protection. (a) Flow cytometric data. (b) Quantitative results. COM concentration: 200 *μ*g/mL; adhesion time: 1 h. Compared with the DC group, ^∗^*p* < 0.05, ^∗∗^*p* < 0.01.

Flow cytometry was used to quantitatively detect the difference in cell adhesion to nano-COM crystals before and after PYP preprotection (Figure 11). The nano-COM crystals were incubated with cells at 4°C. At this temperature, the endocytosis behavior of cells was inhibited, but crystal adhesion was unaffected [20, 21]. The percentage of cells adhering to the crystals was measured by flow cytometry, and cells presenting FITC signals were regarded as cells with adherent crystals (Figure 11(a)). The percentage of cells adhering to crystals in the normal group was 11.05%, which was significantly lower than that in the injured group (51.05%). However, the percentage of cells adhering to crystals in the PYP protection group was 22.71%–44.76% (Figure 11(b)), and the percentage of cells adhering to crystals in the PYP4 protection group was the lowest (22.71%).

## 4. Discussion

### 4.1. PYPs Preprotect HK-2 Cells from Oxidative Damage

The damage of renal epithelial cells is the main reason for kidney stone formation, and the addition of exogenous antioxidants may reduce the oxidative damage of cells. Antioxidants such as vitamin E [22] and seaweed polysaccharides [23] can effectively protect kidney cells from oxidative stress mediated by oxalic acid in vitro. PYP is a polysaccharide with high sulfate content and strong antioxidant capacity. As such, we evaluated the protective effects of PYPs with different Mws on oxalic acid-induced oxidative damage of HK-2 cells. The cell viability test results and cell morphology observation showed that PYP preprotection could reduce the damage of oxalic acid to cells (Figures 1 and 2).

The excessive production of ROS is the main mechanism that causes cell damage. Oxalic acid causes toxicity to cells, leading to the generation of ROS in cells (Figure 3). The production of ROS can cause oxidative stress in cells, leading to the dysfunction of normal cells. ROS primarily occurs in the mitochondria of cells; thus, ROS is the direct cause of the decrease in ΔΨm. Excessive ROS can increase the permeability of the mitochondrial membrane and depolarize the mitochondria, resulting in the decrease of ΔΨm. Our results indicated that cells preprotected by PYPs have reduced ROS production and decreased ΔΨm, thereby reducing oxidative damage caused by oxalic acid.

Oxidative stress can also lead to DNA damage, thereby causing cell cycle retention [24]. Cells in the oxalic acid-treated group experienced S phase retention (Figure 5(b)). The specific inhibition caused DNA damage, leading cells to enter the death procedure [25]. Therefore, mitochondrial dysfunction and DNA damage activate apoptosis, leading to cell apoptosis (Figure 6). The cells protected by PYPs gradually reduced the number of S phase cells and the number of apoptosis. This result is consistent with previous studies, and similar results were obtained in SRA01/04 cells treated with *Lycium barbarum* polysaccharides [26].

**Figure 12:**
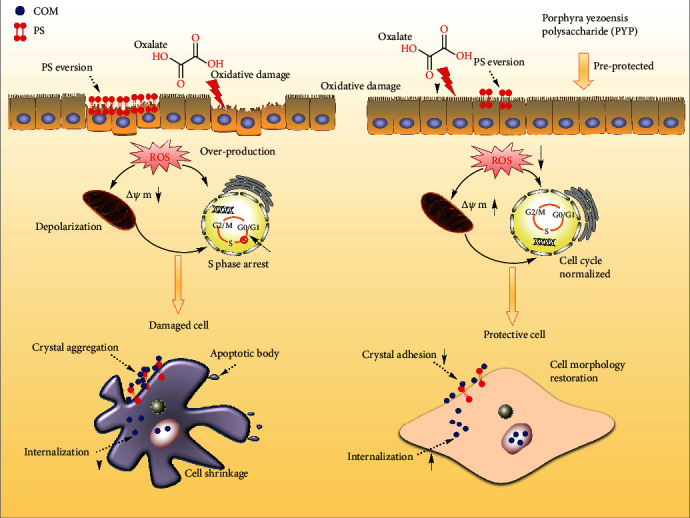
Mechanism diagram of protection of HK-2 cells by PYPs with different molecular weights.

Based on the abovementioned research results, we propose the following causes of the protective effect of PYPs on HK-2 cells (Figure 12): oxalic acid can cause oxidative damage of cells and intracellular ROS generation and lead to mitochondrial dysfunction and S phase cell cycle retention, thereby inducing cell apoptosis. However, after PYPs preprotect the cells, PYPs effectively improved the ability of HK-2 cells to resist the oxidative damage mediated by oxalic acid and reduced the generation of ROS in the cells, the damage to the mitochondria, and the number of cells staying in the S phase and apoptotic cells.

The protective effect of PYPs on HK-2 cells enhances the ability of cells to resist exogenous damage and inhibits changes in cell morphology, apoptosis, and PS eversion, which can increase the resistance of cells to adhered crystals and reduce the risk of crystal aggregation on the cell surface, thereby inhibiting the formation of kidney stones.

### 4.2. PYP Protection Reduces Adhesion to COM Crystals on Cells and Promotes Crystal Endocytosis

Normal renal tubular epithelial cells have a complete structure and function, and few active sites are found on the cell surface, which adhere to urine microcrystals; thus, such cells can resist the adhesion of urine microcrystals. Cell damage is the primary condition for crystal adhesion [27, 28]. The mechanism diagram of protection of HK-2 cells by PYPs with different molecular weights is shown in Figure 12. Our results indicated that oxalic acid can cause damage to cells, destroy the tight junctions between cells (Figure 2), change the polarity of the cell membrane surface, migrate cell membrane components (such as PS) to the cell surface (Figure 7), and induce a large number of negatively charged crystal adhesion molecules such as osteopontin [29], hyaluronic acid, and CD44 on the cell surface [30]. These negatively charged macromolecules can adhere to positively charged surfaces of COM crystals, thereby promoting the adhesion of crystals to cells (Figures 10 and 11). The crystals adhering to the cell surface can further damage the cells and increase the adhesion of the crystals. However, the cells preprotected by PYPs have effectively improved their ability to resist oxidative damage; the degree of cell damage is reduced, and the adhesion sites on the cell surface are reduced, thereby reducing the adhesion of cells to crystals (Figure 11).

However, cells can reduce the damage caused by the adhered crystals through endocytosis [31]. As the endocytosed crystals enter the lysosomes (Figure 8), the crystals dissolve in the acidic environment inside the lysosomes and are expelled from the body [32]. Moreover, as the endocytosed crystals in cells depend on the integrity of the cell membrane structure and function, the endocytosis of damaged cells will be weakened, and crystals entering the lysosomes will be decreased (Figure 9). However, the ability of PYP-protected cells to engulf crystals is enhanced, and the number of crystals entering the lysosomes is increased.

The protection of renal epithelial cells with PYPs can not only reduce the adhesion of crystals on the cell surface but also improve the ability of cells to engulf crystals. These aspects can reduce the risk of adhesion and aggregation of crystals on the cell surface, thereby reducing the risk of kidney stone formation.

### 4.3. PYP with Low Mw Has Good Protective Ability on Cells

The biological activity of polysaccharides is affected by their structural characteristics, including the Mw and functional group content of polysaccharides [33]. Only when the Mw of polysaccharides is within a certain range can it exert the best biological activity, and for polysaccharides with different properties, the Mw range for exerting the best activity is different. Under normal circumstances, degraded polysaccharides can better exert their biological activities. Xing et al. [34] confirmed that the scavenging effect of low-Mw chitosan (9 kDa) on hydroxyl radicals is stronger than that of high-Mw chitosan (760 kDa). Zhao et al. [35] demonstrated that low Mw of sulfated polysaccharides from *Laminaria japonica* has a strong antioxidant effect, which has a protective effect on liver injury induced by CCl_4_ and D-GalN in mice.

The results showed that PYP with low Mw has a strong ability to inhibit HK-2 cells from oxidative damage; thus, PYP4 shows the strongest ability to inhibit COM crystal adhesion and promote endocytosis of COM crystals. The inhibition of HK-2 cells may be related to the following factors: polysaccharides with larger Mw and steric hindrance, overlapping polysaccharide chains, stronger intramolecular hydrogen bond force, tighter structure, and weaker ability to contact with cells, thereby preventing the active groups from exerting their effect [36]. However, the Mw of polysaccharides decreases; polysaccharides have a higher degree of freedom, increased solubility in vivo, and more exposed acidic groups (-OSO_3_-) and can easily enter the cells to react directly with free radicals, thereby increasing the antioxidant capacity of cells. Some studies have also shown that low-Mw polysaccharides have more terminal hydroxyl groups, which are conducive to scavenging free radicals [37]. Therefore, PYP4 with the lowest Mw shows the strongest biological activity.

## 5. Conclusions

Four PYPs with Mws of 576.2, 105.4, 22.47, and 3.89 kDa can protect HK-2 cells from oxidative damage by oxalic acid, and the lower the Mw, the stronger the protective ability of polysaccharides. Under the protection of PYPs, cell vitality increased; cell morphology improved; the ROS level decreased; mitochondrial membrane potential increased; S phase cell arrest was inhibited; and the apoptotic cell rate, PS exposure, and the amount of COM crystals adhering to the cell surface decreased, whereas the amount of endocytosed crystals increased. These results indicated that PYP4 could reduce the risk of calcium oxalate kidney stone formation and might be a potential drug for preventing kidney stones.

## Data Availability

All the data supporting the results were shown in the paper and can be available from the corresponding authors.
